# A Greatly Under-Appreciated Fundamental Principle of Physical Organic Chemistry

**DOI:** 10.3390/ijms12128316

**Published:** 2011-11-28

**Authors:** Robin A. Cox

**Affiliations:** Formerly Department of Chemistry, University of Toronto, 80 St. George St., Toronto, ON, M5S 3H6, Canada

**Keywords:** reaction mechanism, intermediate, lifetimes, excess acidity correlations

## Abstract

If a species does not have a finite lifetime in the reaction medium, it cannot be a mechanistic intermediate. This principle was first enunciated by Jencks, as the concept of an enforced mechanism. For instance, neither primary nor secondary carbocations have long enough lifetimes to exist in an aqueous medium, so S_N_1 reactions involving these substrates are not possible, and an S_N_2 mechanism is enforced. Only tertiary carbocations and those stabilized by resonance (benzyl cations, acylium ions) are stable enough to be reaction intermediates. More importantly, it is now known that neither H_3_O^+^ nor HO^−^ exist as such in dilute aqueous solution. Several recent high-level calculations on large proton clusters are unable to localize the positive charge; it is found to be simply “on the cluster” as a whole. The lifetime of any ionized water species is exceedingly short, a few molecular vibrations at most; the best experimental study, using modern IR instrumentation, has the most probable hydrated proton structure as H_13_O_6_^+^, but only an estimated quarter of the protons are present even in this form at any given instant. Thanks to the Grotthuss mechanism of chain transfer along hydrogen bonds, in reality a proton or a hydroxide ion is simply instantly available anywhere it is needed for reaction. Important mechanistic consequences result. Any charged oxygen species (e.g., a tetrahedral intermediate) is also not going to exist long enough to be a reaction intermediate, unless the charge is stabilized in some way, usually by resonance. General acid catalysis is the rule in reactions in concentrated aqueous acids. The Grotthuss mechanism also means that reactions involving neutral water are favored; the solvent is already highly structured, so the entropy involved in bringing several solvent molecules to the reaction center is unimportant. Examples are given.

## 1. Introduction

In recent years, the study of the mechanisms of organic reactions has been considerably enhanced by the study of putative reaction intermediates [1], often under conditions in which the species are stable enough for spectroscopic examination. For instance, carbocations and other species have been studied extensively in superacid media by Olah and his colleagues [2–4]. However, if a species is to be a reaction intermediate, it has to be stable enough to have a lifetime of at least a few molecular vibrations **under the reaction conditions**, say greater than 10^−13^–10^−14^ s [5]. Jencks pointed this out a number of years ago now [6], as the concept of an “enforced mechanism”; if a species cannot exist under the reaction conditions a mechanism involving it is impossible, and an alternate one is “enforced”.

At the time Jencks wrote his review [6] not a lot was known about the lifetimes of putative reaction intermediates. However, more is known now, and although it is still not easy to apply, the author believes that much more attention has to be paid to what I might call the “Jencks Principle”. For instance, it is certain that primary carbocations cannot exist in a primarily aqueous medium [7], although mechanisms involving them are still occasionally proposed [8]. It is now apparent that this is true of secondary carbocations too [9,10]. In some (but not all) textbooks one still sees mention of “mixed S_N_1 and S_N_2” mechanisms involving secondary substrates [11], due primarily to the early work of the Hughes and Ingold school [12,13], which has since been discredited [13]. It is now well established that secondary substrates react by an S_N_2 process [14], for instance as shown in Scheme I, although for the example shown [15,16] the specific mechanism given is still speculative. The scheme is drawn this way in consequence of the observation that hydroxide ion does not add to carbonyl groups directly, but instead attacks a water molecule which does the actual addition [17–19]. Enough kinetic evidence to prove or disprove this probably exists [15,16], and work to do this is underway [20]. Hydroxide ion is not very reactive. It is less solvated, and hence much more reactive, in alcohol solvents, and in pure DMSO its reactivity is increased by some twelve orders of magnitude [21].

For the mechanisms of reactions in aqueous media, far more important is the observation that species such as H_3_O^+^ (usually called the Eigen cation [22]), H_5_O_2_^+^ (usually called the Zundel cation [23,24], although also strongly preferred by the school of Vinnik and Librovich at the Institute of Physical Chemistry in Moscow [25]), H_9_O_4_^+^ (first postulated by Bell [26], but often (mistakenly) also called the Eigen cation) and the many others which have been proposed [27] (not that there has ever been any believable experimental evidence for any of them [28,29]) do not have lifetimes long enough to exist. Although far less work has been done, recent studies show that HO^−^ cannot exist as such in water either [30–32]. Recent very high-quality IR measurements on acid solutions [33,34] show that the only structure that has any kind of real existence in them is the proposed H_13_O_6_^+^ [35], shown in Scheme II [34], but even this has a very short lifetime; the authors state [36]: “The lifetime of the five central protons is close to the time of their vibrational transitions. In ~70% of these cations it is shorter than the time of normal vibrations and the IR spectrum degenerates to a continuum absorption”. In addition, in several modern theoretical calculations on proton clusters containing many water molecules it is found not to be possible to isolate the positive charge, it is simply “on the cluster” as a whole [37].

Consequently, we may only speak of “H_aq_^+^” and “HO_aq_^−^” as being reactants [28–34]. The Grotthuss chain transfer process along hydrogen bonds in water simply ensures that a proton or a hydroxide ion is available instantaneously where or when it is needed. (This is such a widely accepted transport mechanism in water that specific references to it are difficult to find. The original is [38]). This has all kinds of consequences for reaction mechanisms in predominantly aqueous acidic and basic media. For instance, we can no longer speak of “general” and “specific” acid and base catalysis of reactions. Far better to speak of “pre-equilibrium proton transfer”, in the case of reactions that involve the formation of a stable ionized intermediate (usually by resonance), and of “proton transfer as part of the rate-determining step”, in the other cases. Several examples follow.

The highly structured nature of liquid water [39] also ensures that reaction mechanisms involving several water molecules acting in concert are also favored. The entropy involved in bringing water molecules into the right positions is not a concern as the structure is already there, and the Grotthuss process ensures that all proton transfers are essentially instantaneous. Several examples of reactions of this type will be given as well.

## 2. Results and Discussion

### 2.1. General Acid Catalysis in Strong Acid Media

As far as the common strong acids HCl, HClO_4_ and H_2_SO_4_ are concerned, the only acid species present is “H_aq_^+^” under normal conditions, and reactions in all of them therefore ought to proceed at the same rate at the same acid concentration [40].

Sulfuric acid is the only one that can be used from 0 wt% to 100 wt%, the dilute solution containing H_aq_^+^. Above the 1:1 H_2_O:H_2_SO_4_ molecular ratio (84.48 wt%) there is, of course, no free water present, but the solution now contains catalytically active undissociated sulfuric acid molecules. Above 99.5 wt% autoprotolysis becomes important, with the very strong acid species H_3_SO_4_^+^ present as a possible catalyst as well [41]. I found catalysis by both of the latter species as far back as 1974 in the Wallach rearrangement of azoxybenzene, Scheme III [41–43]. This reaction has been extensively reviewed [44,45], so I will not say much about it here. The species which are stable enough to exist in the reaction solution are indicated in the Scheme; interestingly, both of them have been observed experimentally under stable ion conditions [4]. Theoretical calculations have shown the dicationic species to have the structure shown, with little communication between the two halves of the molecule [42].

Interestingly H_aq_^+^ is not a strong enough acid species to catalyze the reaction, only catalysis by H_2_SO_4_ and by H_3_SO_4_^+^ being observed [41,44]. The reaction does not work in HClO_4_, a stronger acid system in *H*_0_ terms but only containing H_aq_^+^ with no undissociated HClO_4_ molecules present [45,46]. It does go in pure FSO_3_H and ClSO_3_H, both being quite strong acid species [46].

Another case of general acid catalysis was observed in the hydrolysis of several ethyl thiolbenzoates in sulfuric acid at concentrations above 60 wt%, where catalysis by H_aq_^+^ was observed, catalysis by undissociated H_2_SO_4_ molecules taking over above 80 wt% in concentration [47], Scheme IV.

### 2.2. Ether Hydrolyses

The hydrolyses of trioxane and similar molecules in dilute acid have been taken by many authors (even by myself [48]) to be typical A1 processes, protonation followed by rate-determining breakup of the protonated intermediate. However, if H_3_O^+^ cannot exist in water, other species with positive charge on oxygen which is not resonance-stabilized are not going to be capable of existence either. This means that the mechanism of the hydrolysis of trioxane is going to be that given in Scheme V. (Scheme V shows the breakup to three formaldehyde molecules taking place all at once, but a similar stepwise breakup is of course also possible.)

There is plenty of kinetic data on this reaction in several different acid media available for analysis [49]. The preferable method to use for this is the excess acidity correlation analysis [48], which is used here. The applicable rate equation is shown as Equation 2.

(1) $$k_{\psi }C_{\text{S}}=k_{0}a_{\text{S}}a_{{\text{H}}_{2}\text{O}}a_{{{\text{H}}^{+}}_{\text{aq}}}/f_{‡}=k_{0}C_{\text{S}}a_{{\text{H}}_{2}\text{O}}C_{{{\text{H}}^{+}}_{\text{aq}}}\frac{f_{\text{S}}\;f_{{{\text{H}}^{+}}_{\text{aq}}}}{f_{‡}}$$

(2) $$\text{log}\;k_{\psi }-\text{log}\;C_{{{\text{H}}^{+}}_{\text{aq}}}-\text{log}\;\;a_{{\text{H}}_{2}\text{O}}=\text{log}\;\;k_{0}+m^{‡}\;m^{*}\;X$$

**Figure 1:**
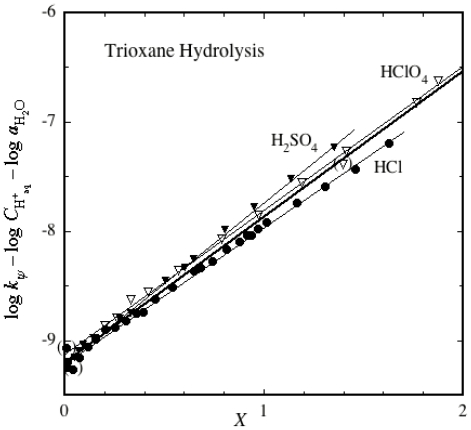
Excess acidity plot for trioxane hydrolysis in dilute H_2_SO_4_, HCl and HClO_4_.

Here the observed rate constants are *k_ψ_* [49], the medium-independent rate constant (*i.e.*, the rate constant in the aqueous standard state) is *k*_0_, the proton concentration is *C*_H_aq_^+^_, the water activity is *a*_H_2_O_ and the excess acidity is *X*, all available data for all three acid systems [48]. The slope parameters m* and *m*^‡^ describe the behavior of the protonated substrate and the transition state as the acidity changes, necessarily combined here [48]. Plots according to Equation 2 are given in Figure 1.

As can be seen, the plots for all three acids are accurately linear. For illustration purposes a thick line is given for all of the data combined, slope 1.333 ± 0.022, intercept –9.198 ± 0.018, correlation coefficient 0.993 over 54 points. However, the points for the three individual acids fall (very accurately, correlation coefficients 0.9990 in HCl, 0.9994 in HClO_4_, 0.9994 in H_2_SO_4_) on slightly different lines, which undoubtedly reflects the fact that the water activities for the three acids are not known equally well. Water activities in the aqueous sulfuric acid medium [50] are very accurately known [51], but this is not the case for HCl [52–54] and, particularly, HClO_4_ [55–58]. All of the plots fit the appropriate lines more closely than was previously found by treating the process as a traditional A1 reaction [48].

If this process is really a case of general acid catalysis, rates measured in aqueous buffer systems should show this. Trioxane hydrolysis is too slow a reaction to have been studied in this way, but the closely related hydrolysis of paraldehyde (the acetaldehyde trimer) is much faster [48], and evidence for general acid catalysis has indeed been found [59,60], although this fact does not seem to be widely known (or has been ignored). A plot like Figure 1 can also be drawn for paraldehyde, but the kinetics cover a much smaller acidity range, and the scatter is bad.

Another ether system for which kinetic results are available [61] is the hydrolysis of diethyl ether at high temperatures and high acidities in aqueous sulfuric acid. The mechanism proposed here is shown in Scheme VI.

**Figure 2:**
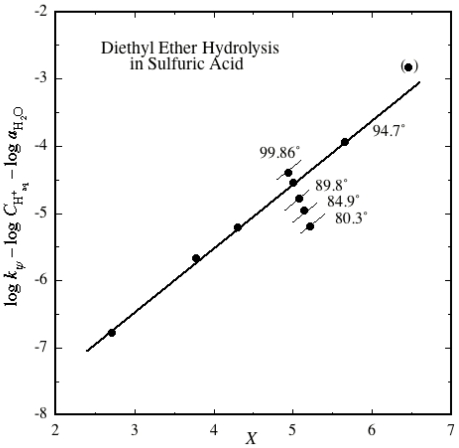
Excess acidity plot for the hydrolysis of diethyl ether in relatively concentrated H_2_SO_4_, at several temperatures.

This is essentially the same mechanism as that shown in Scheme V, and the same excess acidity rate equation, Equation 2, applies. In sulfuric acid this mechanism is only going to apply as long as there is free water available, *i.e*., not above a concentration of 85.48 wt%. Above this acidity another well-characterized mechanism takes over [61], involving a much faster direct reaction between the diethyl ether and SO_3_, which is available for reaction above this acidity. Thus in an excess acidity plot one would expect linearity below 85.48 wt%, and an upward deviation above this point. This is exactly what is observed, as Figure 2 illustrates.

The topmost point in Figure 2 is at an acidity of 90 wt%, and deviates upwards as expected. (In the original paper [61] a plot of log rate constant against acidity curves downward over the acidity region which gives linearity here.) The m*m^‡^ slope is 0.949 ± 0.015, and as different temperatures are available, the activation parameters for the reaction can be calculated: Δ*H*^‡^ = 32.8 ± 1.4 kcal·mol^−1^; Δ*S*^‡^ = −12.4 ± 4.7 cal·deg^−1^·mol^−1^, both perfectly reasonable numbers. (They only concern the substrate, as *X*, log *C*_H_aq_^+^_and log *a*_H_2_O_ have all been corrected to the reaction temperature, as before [48].) The correlation coefficient is 0.9993.

Figures 1 and 2 constitute strong evidence in favor of the mechanisms given here. Interestingly, it does not matter whether the substrate can be considered to be primarily protonated at the acidity of the reaction or not; oxygen-protonated species in which the charge cannot be delocalized are not going to be reaction intermediates as their lifetimes are too short! When the charge **can** be delocalized, intermediate lifetimes are much longer. For instance, the methoxymethyl cation, where the charge is delocalized over carbon and oxygen, is calculated to have a lifetime of about 1 ps [62], which, although short, is quite long enough for it to be a reaction intermediate.

### 2.3. Amide Hydrolyses

Benzamides, and presumably other suitable amides, have two hydrolysis mechanisms [63]. In weakly acidic aqueous H_2_SO_4_ media, a pre-equilibrium proton transfer gives a stable delocalized protonated amide intermediate, to which water adds; see Scheme VII. From this a neutral tetrahedral intermediate is formed directly; charged ones cannot exist in an aqueous medium. (Log rate constants, corrected for incomplete amide protonation, are linear in the log water activity, slope two. Molarity-based water activities must be used for consistency with the other species concentrations, rather than the listed mole-fraction-based ones [48].)

In more strongly acidic media the mechanism changes [63]; the kinetics show a second, concerted, proton transfer taking place, giving an acylium ion which is stable under the reaction conditions, and that two water molecules are involved [63]. This mechanism is a bit tricky to draw, but I have made an attempt in Scheme VIII. Since an acylium ion is involved, this mechanism would only occur for those amides capable of giving stable ones, primarily benzamides. For other types of amide evidence is lacking; amides are particularly stable and their acid hydrolysis is very slow and quite difficult to study. The catalyzing acid is given as H_aq_^+^; presumably in H_2_SO_4_ media stronger than ~85 wt% the catalyst would be undissociated H_2_SO_4_, see above [63].

### 2.4. Ester Hydrolyses

At acidities below ~85 wt% the mechanisms of these processes are similar to those for benzamides [63] (and benzimidates [64]) as shown in Scheme IX [64], which differs from Scheme VII for amides in that the neutral tetrahedral intermediate does not contain a nitrogen atom, and so it is susceptible to ^18^O-exchange, which is observed [65]; it is essentially not found in amide hydrolysis [66].

In the strong acid region, above ~85 wt% H_2_SO_4_, other mechanisms take over. If the substrate contains a group capable of forming a stable carbocation, e.g., a benzylic or a tertiary group, this can leave directly from the protonated ester, and this can be the preferred mechanism at acidities much lower than 85 wt% H_2_SO_4_ [67,68]. This is shown in Scheme X.

For other esters in strong acid an additional proton transfer is probably involved, to give an acylium ion; the previously proposed [67,68] “proton switch” mechanism is probably wrong. This again is quite difficult to draw, but I have made an attempt in Scheme XI. This mechanism is not yet established, but work is underway to do this [20].

In basic media, it is becoming increasingly apparent that hydroxide ions do not themselves add directly to carbonyl groups, but that HO_aq_^−^ removes a proton from a water molecule which then adds to the carbonyl, the result being a neutral tetrahedral intermediate [17–19]. Heavy-atom isotope effect studies make this appear even more likely [69]. Since the process is reversible, extensive oxygen exchange into the substrate is observed as well [70,71]. The most probable mechanism is given here as Scheme XII. Formation of a neutral intermediate ensures that the negative charge is dispersed into the solvent. Electronegative oxygen is certainly more able to support a negative charge than a positive one, but the principle of having any charge, positive or negative, dispersed as widely as possible ensures that all tetrahedral intermediates formed in either acidic or basic processes would be neutral. Species that are represented by various authors as T^+^, T^−^, T^±^ and, especially, T^2−^ do not exist in aqueous media.

### 2.5. Mechanisms Involving Chains of Water Molecules

There are quite a number of these known now. The principles seem to be that if a reaction can be achieved without any charge transfer taking place it is favored, and that reactions involving chains of water molecules are favorable because the structure necessary for reaction essentially already exists; water molecules do not have to be moved into position, which is unfavorable entropically. For instance, acylimidazoles hydrolyze by forming a tetrahedral intermediate directly, Scheme XIII [72]. Incidentally, this work showed that the excess acidity correlation analysis works well even for reactions that are not acid-catalyzed [72].

I proposed a mechanism for the hydrolysis of nitramide in neutral water on the basis of nothing but its elegance [73], and was gratified that detailed modern theoretical calculations, in the gas-phase and also in solution [74], showed that it was in fact correct. This is shown in Scheme XIV.

The hydrolyses of acid chlorides and acid anhydrides are fast reactions which have not received a lot of attention. Several mechanisms have been proposed [75–78], but the latest research would indicate that the actual mechanism may well be a simple cycle involving water as well, Scheme XV [78].

## 3. Conclusions

1. If a species does not have a finite lifetime in the solution in which the reaction is performed it cannot be a reaction intermediate. No primary or secondary carbocations in aqueous media; only T^0^, no T^+^, T^−^, T^±^ or T^2−^ tetrahedral intermediates.
2. Positive or negative charge, if present, will be as delocalized as possible during the reaction, especially in reaction intermediates, often into the aqueous solvent. A highly electronegative atom like oxygen is simply not going to support a positive charge all by itself. O^+^ is almost as unlikely as F^+^!
3. Also, reactions will be unimolecular, as far as possible, for entropic reasons (S_N_1 favored over S_N_2); however, mechanisms involving chains of water molecules are favored in aqueous media thanks to the highly structured nature of water and the Grotthuss process.

There are a number of philosophical implications. Many years ago chemists weaned themselves from using “H^+^” as a reactant, once it was pointed out that free protons are only stable in a hard vacuum. Now we are going to have to wean ourselves from using “H_3_O^+^” or “HO^−^” as reactants in aqueous solution as well. Of course these species do exist, under special circumstances. In sulfuric acid above the 1:1 mole ratio point (~85 wt%) all the remaining water is present in the form H_3_O^+^. The perchloric acid hydrate sold as a solid in glass vials is H^3^O^+^·ClO_4_^−^ (and is pretty dangerous stuff!). The terms to use are “H_aq_^+^” and “HO_aq_^−^”.

We are going to have to cease using the terms “general” and “specific” acid and base catalysis. Much to be preferred, I think, is to refer to “pre-equilibrium proton transfer” when an intermediate that is stable under the reaction conditions is formed in a first fast step, and to “concerted with proton transfer”, or something similar, when the proton transfer is involved in the rate-determining step, as in many of the examples discussed above.

Very recently some common organic reactions have begun to be studied in liquid ammonia as a solvent, rather than in water [79,80]. It is going to be very interesting to compare the mechanisms of the same reaction in the two different solvents.

## Figures and Tables

**Scheme I:**
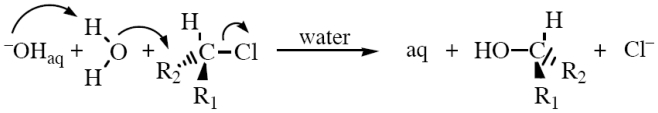
S_N_2 substitution of a secondary alkyl halide by hydroxide ion.

**Scheme II:**
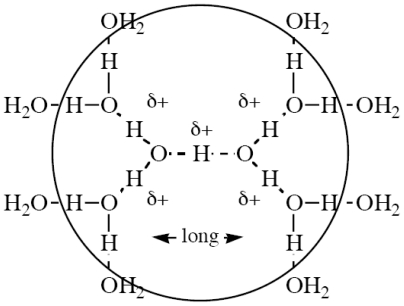
Structure of the only solvated proton species detected in water.

**Scheme III:**
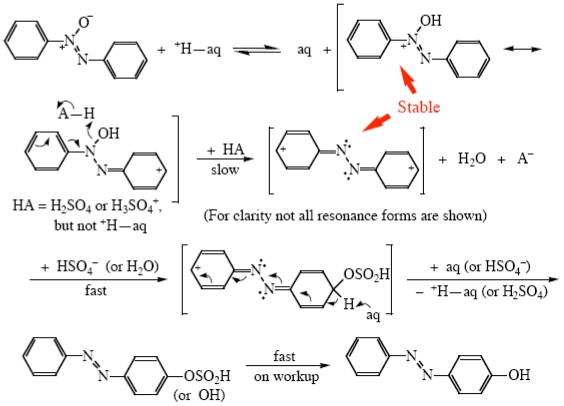
Wallach rearrangement of azoxybenzene in sulfuric acid.

**Scheme IV:**
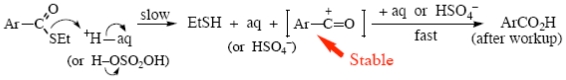
Hydrolysis of ethyl thiolbenzoates in sulfuric acid.

**Scheme V:**
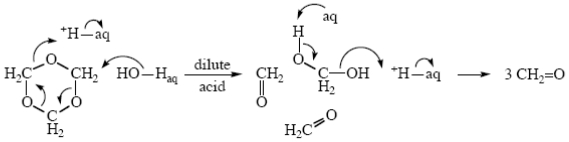
Hydrolysis of trioxane in dilute acid.

**Scheme VI:**
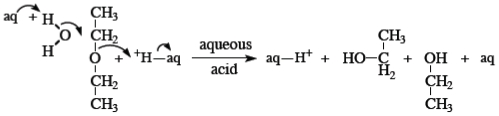
Acid hydrolysis of diethyl ether.

**Scheme VII:**
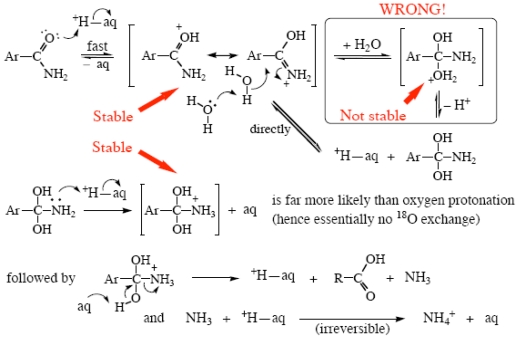
Acid hydrolysis of benzamides in <60 wt% H_2_SO_4_.

**Scheme VIII:**
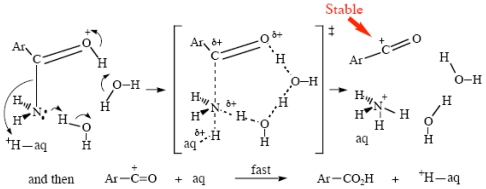
Acid hydrolysis of benzamides in >60 wt% H_2_SO_4_.

**Scheme IX:**
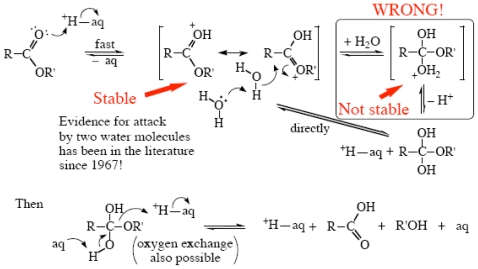
Acid hydrolysis of esters in <85 wt% H_2_SO_4_.

**Scheme X:**
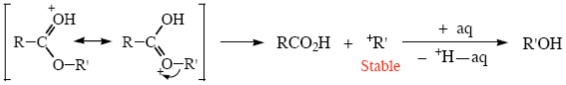
Acid hydrolysis of esters capable of forming carbocations.

**Scheme XI:**
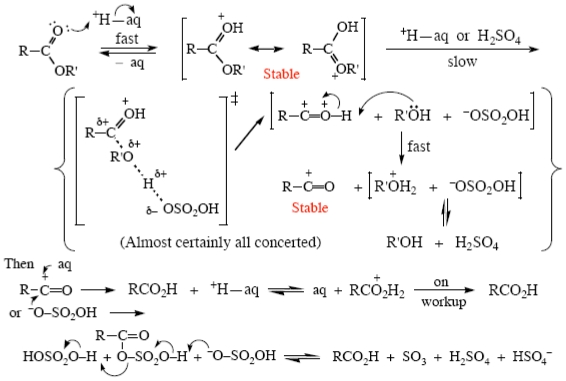
Acid hydrolysis of other esters in >85 wt% H_2_SO_4_.

**Scheme XII:**
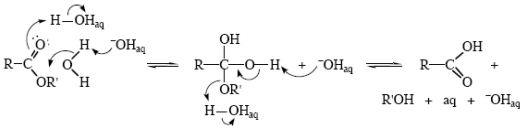
Basic ester hydrolysis.

**Scheme XIII:**
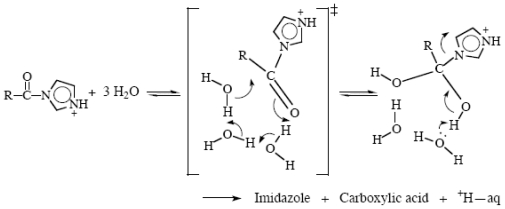
The mechanism of hydrolysis of acylimidazoles in water.

**Scheme XIV:**
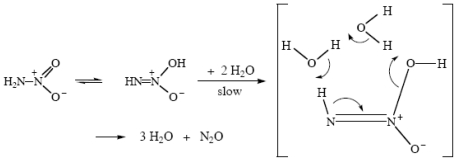
Nitramide hydrolysis in neutral water.

**Scheme XV:**
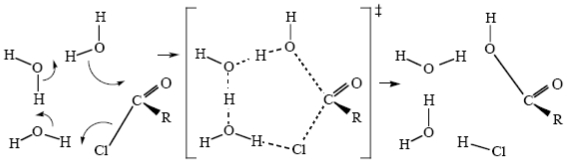
A possible mechanism for acid chloride hydrolysis in water.
